# Inequalities in Crowdfunding for Transgender Health Care

**DOI:** 10.1089/trgh.2018.0044

**Published:** 2019-03-06

**Authors:** Chris A. Barcelos, Stephanie L. Budge

**Affiliations:** ^1^ Department of Gender and Women's Studies, University of Wisconsin-Madison, Madison, Wisconsin.; ^2^ Department of Counseling Psychology, University of Wisconsin-Madison, Madison, Wisconsin.

**Keywords:** access to care, crowdfunding, inequalities, medical gender affirmation, sex reassignment surgery, transgender

## Abstract

**Purpose:** An emerging body of research analyzes the scope, ethics, and inequalities of web-based crowdfunding to raise money for medical expenses related to illness or injury. To date, little research has investigated how transgender communities utilize crowdfunding expenses related to gender affirming medical care.

**Methods:** Using GoFundMe.com, we created a data set of 391 crowdfunding campaigns for gender-affirming care created from 2012 to 2016. In addition to descriptive statistics of recipient demographics and campaign financials, we conducted hierarchical multiple regression analyses to determine the factors associated with financial outcomes of fundraising for medical expenses.

**Results:** Findings indicate that the majority of campaigns were used to fund chest surgeries among young, white, binary-identified trans men in the United States. Few campaigns met their fundraising goal. Being a trans man whose Facebook community shares the crowdfunding campaign is predictive of meeting a higher percentage of the fundraising goal, whereas being a trans woman whose Facebook community shares the campaign is predictive of raising more money.

**Conclusion:** The use of crowdfunding for gender affirming highlights the difficulties that transgender persons face with using private and public health care programs to fund medically necessary care. Health care providers should exercise caution in recommending crowdfunding as a viable strategy to raise money for out-of-pocket costs.

## Introduction

Medical crowdfunding, the practice of soliciting donations from social networks to pay for health care, has become so ubiquitous that one journalist referred to it as the “sad, dark, future of health care.”^1^ Platforms such as GoFundMe.com and YouCaring.com enable individuals seeking financial assistance to solicit donations, to post photos and treatment updates, and to share the appeal throughout their social networks. In the United States, medical bills are the number one cause for debt and bankruptcy,^2^ and despite the (constantly threatened) Affordable Care Act (ACA), 27.6 million people remain uninsured.^3^

The inequalities in accessing and paying for medical care are especially germane to transgender individuals, who face a variety of exclusionary policies and practices within the health care system. At an individual level, transgender people experience discrimination from health care providers ranging from refusal of care to physical violence.^4,5^ At a structural level, trans people face issues such as health insurance policies with categorical exclusions for gender-affirming care and a lack of trans-competent medical providers.^6–11^

Research in a variety of social and biomedical disciplines has demonstrated that access to gender-affirming medical care is associated with better health outcomes. Access to gender-affirming care is associated with improvements in psychological health^12–16^ and quality of life,^15,17–19^ reduced HIV incidence,^16,20^ decreased viral load,^21^ decreased substance use,^22^ and increased engagement in preventive health services.^23^ However, trans people face numerous and extraordinary barriers in obtaining gender-affirming care.^7,9^ Transgender people are more likely than cisgender people to be uninsured,^4,24^ face a shortage of quality, accessible providers,^4^ and experience provider discrimination.^4,5,8^ Notably, exclusions for gender-affirming care in insurance policies mean that many trans people pay out of pocket for the same services that a plan would cover for a cisgender patient,^7,25^ which can range from approximately $5,000 to $100,000 U.S. dollars.^26^ Even in states with nondiscrimination policies that prohibit trans-exclusionary health insurance policies, trans people experience difficulty in accessing care due to frequently changing rules, difficulty in navigating insurance plan bureaucracies, and a lack of available providers.^4,9–11^ On top of this, transgender populations earn lower wages, are disproportionately of low income, and experience high levels of employment discrimination.^27^

Crowdfunding is a strategy to raise money for out-of-pocket medical expenses used by transgender and cisgender people alike. Although there is a growing body of scholarship that investigates ethics, efficacy, privacy, and inequalities related to crowdfunding,^28–30^ little research has analyzed how transgender individuals utilize crowdfunding to raise money for medical gender affirmation. Existing studies only examine small samples of campaigns or use interview-based research with trans people who used crowdfunding and thus cannot assess the distribution and determinants of trans medical crowdfunding.^31,32^

## Methods

The first author created a cross-sectional data set composed of the demographics, financials, social media shares, narratives, and photos of medical gender affirmation fundraisers on the GoFundMe.com web site. We selected this site because it is the largest online crowdfunding site^33^ and appears popular in transgender communities. Because these are publicly available data that are accessible without a creating an account, they are not considered human subjects data as defined by institutional review boards. Nevertheless, we have taken steps to protect the privacy of fundraisers by only presenting data in the aggregate. First, in June 2016, the first author searched the web site for all active campaigns matching the term “trans.” This search resulted in 493 campaigns. Numerical data on fundraising goals, amount raised, the recipient's zip code, and the number of Facebook shares were extracted into a spreadsheet.

Next, inclusion and exclusion criteria were applied, resulting in 410 fundraising campaigns created from March 2012 to May 2016 in six countries. Campaigns met the inclusion criteria if they were an active fundraiser created for a single person to raise money for gender-affirming medical care, including hormone treatment and surgical procedures (any related costs including co-pays, prescriptions, laboratory work, and provider visits). Campaigns for medical expenses not related to medical gender affirmation were excluded (including living expenses, sperm or egg banking, costs associated with legal name changes, gender-affirming clothing/prosthetics such as chest binders, packers, and bra inserts). The purpose of these inclusion and exclusion criteria was to identify campaigns that were specifically intended for out-of-pocket expenses related to gender-affirming care.

In addition to the data on fundraising amounts and social media shares, several variables were created to assess the purposes of the campaigns, the age distribution of the recipients, and the recipients' race, sex assigned at birth, and gender identity. The campaigns of individuals who identified with a gender identity other than trans women or trans men (nonbinary=7; intersex=4, Two Spirit=1) were removed due to the small sample size lending to uninterpretable group results.^a^ The narrative elements of the data set are analyzed in a separate article.^34^

Two hierarchical multiple regression analyses were conducted to determine the factors associated with financial outcomes of fundraising for medical expenses. Independent variables in both analyses included the number of Facebook friends and the number of Facebook shares for the fundraising project. The two separate dependent variables for fundraising outcomes included the percentage of the fundraising goal attained and the actual amount of money raised from the fundraising campaign. Number of donors and transgender identity were included as moderator variables for both Facebook friends and Facebook shares. For each hierarchical regression, race, gender identity, age, and U.S./International were entered as covariates in Step 1. At Step 2, the independent variables were entered to test for main effects. At Step 3, the moderator variables were entered to determine any interaction effects.

## Results

This data set included *N*=391 campaigns. Approximately 90% of campaigns were created by the intended recipient of the funds; the remainder were created by friends, significant others, and/or family members. In this sample, recipients' ages ranged from 15 to 73 years (*M*=23.90; SD=7.27). For gender identity, 70.1% identified as trans men and 29.9% identified as trans women. For race, 11% (*n*=43) were identified as people of color and 84.6% (*n*=331) were identified as white individuals.^b^ Additional demographic information is listed in Table 1.

**Table 1.** Recipient and Campaign Characteristics

	*n*	%
Recipient characteristics		
Trans men	274	70.1
Trans women	117	29.9
People of color	43	11.0
Recipient based in the United States	380	84.9
Northeast	64	16.4
South	106	21.7
Midwest	82	21.0
West	28	7.2
Pacific	52	13.3

	Mean	SD	Range
Campaign characteristics			
No. of Facebook friends	615.79	864.26	3–5000
No. of Facebook shares	202.21	361.47	0–4464
Goal USD	$9,513.00	$11,095.00	$100–78,218
Amount raised	$1,296.20	$2,138.83	$0–$23,746
% of goal	21.12	30.55	0–147
No. of donors	29	80	0–1422
Length of campaign, days	350	290	36–1558

USD, U.S. dollars.

The mean for the number of Facebook friends was 615.79 (SD=864.26, range=3–5000). The mean number of campaign Facebook shares was 202.21 (SD=361.47, range=0–4464). Regarding the dependent variables, the mean percentage raised based on the stated goal was 24.12% (SD=30.55, range=0–147%). The mean fundraising goal was $9,513 (SD=$11,095, range=$100 to $78,218). The mean amount of money raised was $1,296.20 (SD=2,138.83, range=$0 to $23,746). The mean number of donors to a campaign was 29 (SD=80, range=0–1422). Finally, at the time of the data collection, campaigns had been active for a mean of 350 days (SD=290, range 36–1558) (Table 1). The majority of campaigns (61.9%) listed mastectomy with masculinizing reconstruction (“top surgery”) as their primary purpose. The second most common procedures were vaginoplasty (12.8%) and hormone therapy (12.5%) (Table 2). Correlations for all variables are included in Table 3.

**Table 2.** Campaign Purpose

	*n*	%	Mean goal	Mean % raised
“Top surgery”/mastectomy with masculine reconstruction	242	61.9	$6,695.60	27.8
Vaginoplasty	50	12.8	$17,489.00	12.8
Hormones	49	12.5	$8,068.23	25.7
Laser hair removal/electrolysis	11	2.8	$7,668.36	25.7
Facial feminization	9	2.3	$22,715.67	12.7
Breast augmentation	8	2.1	$7,212.50	24.4

Not all campaigns were able to be coded for purpose; data not included for procedures constituting less than 1% of the data set (phalloplasty, hysterectomy, “tracheal shave,” feminization laryngoplasty, orchiectomy).

**Table 3.** Correlation Matrix

Construct	1	2	3	4	5	6	7	8	9
1. Age	1								
2. Trans identity	−0.35^**^	1							
3. Race	−0.15^*^	−0.04	1						
4. Location	0.02	0.06	0.06	1					
5. Facebook friends	0.01	−0.03	0.16^**^	0.03	1				
6. Facebook shares	0.03	−0.05	0.10	−0.09	0.48^**^	1			
7. Donors	0.02	−0.08	0.03	−0.14^**^	0.28^**^	0.74^**^	1		
8. Amount raised	0.08	−0.01	0.04	−0.09	0.22^**^	0.66^**^	0.80^**^	1	
9. Percentage of goal raised	−0.10	0.18^**^	−0.03	0.02	0.07	0.30^**^	0.30^**^	0.58^**^	1

^*^ *p*<0.05, ^**^p<0.01.

### Total U.S. dollars raised

**FIG. 1.**
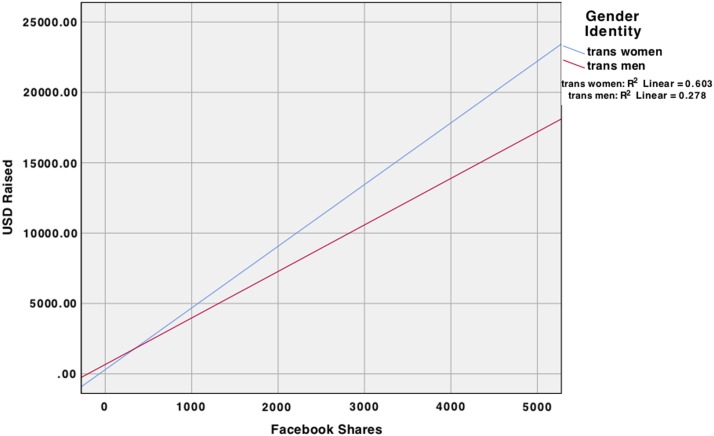
Interaction effect of Facebook shares and gender identity on US dollars raised.

The overall regression analysis (Table 4) of Facebook friends and shares predicting total U.S. dollars raised was significant. The only covariate that was significant, without accounting for other variables in the model, was location—indicating that individuals raised less money internationally (β=−0.18, *p*=0.001), when not taking any social media into account. When covariates and main effects were included, the regression analysis was significant, *F*(7, 182)=70.24, *p*<0.01. Here, the amount of Facebook shares (β=0.23, *p*<0.001) was significant, such that more Facebook shares predicted more funds raised and the number of donors was also significant (β=0.61, *p*<0.001) in the expected direction. When accounting for main effects, age of recipient was significant (β=0.11, *p*=0.01), indicating that older individuals raised more funds. Results also indicated that trans men raised significantly more funds than trans women. An additional 69% (total *R*^2^=0.73) of the variance was accounted for when adding main effects, *F*Δ(3, 182)=154.89, *p*<0.001. When including the moderator variables into the model, the model remained significant—transgender identity moderated the number of Facebook shares when predicting U.S. dollars raised, such that trans women who had more Facebook shares raised more money when compared with trans men who had the same amount of Facebook shares (Fig. 1).

**Table 4.** Hierarchical Multiple Regression Results

	US dollars raised				p
	*R*^2^	Δ*R*^2^	β	*t*	
Step 1	0.04				
Age			0.07	0.89	0.37
Trans identity			−0.04	−0.53	0.59
Race			−0.003	−0.04	0.97
Location			−0.18	−2.53	0.01^**^
*F*(4, 185)=1.94, *p*=0.11					
Step 2	0.73	0.69			
Age			0.11	2.64	0.01^**^
Trans identity			0.11	2.47	0.02^*^
Race			−0.01	−0.16	0.87
Location			−0.01	−0.17	0.86
Facebook friends			−0.04	−0.91	0.36
Facebook shares			0.29	3.97	0.000^**^
Number of donors			0.61	8.66	0.000^**^
*F***Δ**(3, 182)=154.89, *p*<0.001					
Step 3	0.80	0.04			
Age			0.06	1.73	0.09
Trans identity			0.09	2.50	0.01^**^
Race			−0.01	−0.17	0.87
Location			0.01	0.23	0.82
Facebook friends			−0.04	−0.32	0.75
Facebook shares			−0.26	−1.56	0.12
Number of donors			1.37	8.53	0.000^**^
Donors×Facebook friends			0.80	0.88	0.38
Donors×Facebook shares			−1.48	−1.7	0.09
Trans identity×Facebook friends			0.05	0.44	0.66
Trans identity×Facebook shares			0.42	3.05	0.003^**^
*F***Δ**(4, 178)=16.86, *p*<0.001					

^*^ *p*<0.05, ^**^p<0.01.

### Percentage of goal raised

**FIG. 2.**
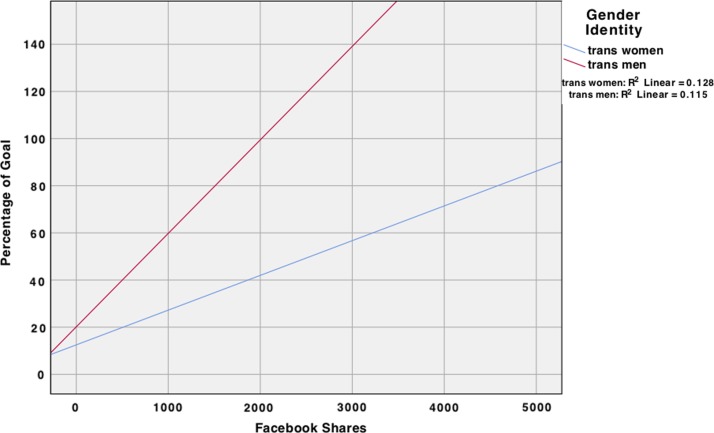
Interaction effect of Facebook shares and gender identity on the percentage of goal raised.

The overall regression analysis (Table 5) of Facebook friends and shares predicting the percentage of goal raised was significant. When we only included covariates in the model, the only covariate that was significant was gender identity—indicating that trans men raised a higher percentage of their stated goal when compared with trans women (β=0.20, *p*=0.01). When we included both covariates and main effects into the model, the regression analysis was significant, *F*(7, 182)=4.64, *p*<0.001. Similar to the previous analysis, the amount of Facebook shares (β=0.31, *p*<0.001) was significant, such that more Facebook shares predicted individuals attaining a higher percentage of their stated goal. Regarding covariates, gender identity remained significant (β=0.24, *p*=0.001). An additional 10% (total *R*^2^=0.15) of the variance was accounted for, *F*Δ(3, 182)=6.95, *p*<0.001, when the main effects were added to the model. When moderators were added to the model, transgender identity moderated the number of Facebook shares when predicting the percentage of goal raised, such that trans men who had more Facebook shares raised a higher percentage of money compared with trans women who had the same amount of Facebook shares (Fig. 2).

**Table 5.** Hierarchical Multiple Regression Results

	Percentage of goal raised				p
	*R*^2^	Δ*R*^2^	β	*t*	
Step 1	0.05				
Age			−0.02	−0.23	0.37
Trans identity			0.20	−2.55	0.59
Race			−0.07	−0.98	0.97
Location			−0.03	−0.43	0.01^**^
*F*(4, 185)=2.65, *p*=0.04					
Step 2	0.15	0.12			
Age			0.02	0.20	0.01^**^
Trans identity			0.26	3.47	0.02^*^
Race			−0.08	−1.16	0.87
Location			0.02	0.32	0.86
Facebook friends			−0.03	−0.43	0.36
Facebook shares			0.31	2.34	0.000^**^
Number of donors			0.03	0.26	0.000^**^
*F***Δ**(3, 182)=6.95, *p*<0.001					
Step 3	0.37	0.22			
Age			−0.07	−1.06	0.09
Trans identity			0.24	3.60	0.01^**^
Race			−0.09	−1.43	0.87
Location			0.05	0.74	0.82
Facebook friends			−0.12	−0.53	0.75
Facebook shares			−0.56	−1.86	0.12
Number of donors			1.56	5.44	0.000^**^
Donors×Facebook friends			−1.13	−0.69	0.38
Donors×Facebook shares			−0.20	−0.13	0.09
Trans identity×Facebook friends			0.10	0.47	0.66
Trans identity×Facebook shares			0.65	2.64	0.003^**^
*F***Δ**(4, 178)=15.27, *p*<0.001					

^*^ *p*<0.05, ^**^p<0.01.

## Discussion

To the best of our knowledge, the analysis presented here is the first large sample study documenting the distribution and determinants of transgender medical crowdfunding. Crowdfunding is a response to health and social inequalities related to a disproportionate burden of ill health and lack of adequate insurance coverage for gender-affirming care. Yet, the little research available indicates that medical crowdfunding does little to remedy these inequalities and may in fact exacerbate them.^28^ Lack of adequate health care coverage is even more pronounced in transgender communities and plays out in trans people's attempts to fund medically necessary gender-affirming care through web-based crowdfunding.

Our analysis revealed several inequalities in crowdfunding for gender-affirming care. First, the majority of recipients were young, white, binary-identified transgender men. This finding indicates that those with most pre-existing social, economic, and racial privilege disproportionately use crowdfunding to raise money for gender-affirming care. Second, similar to Berliner and Kenworthy,^28^ we found that few campaigns reach their goal. This result is consequential given that the access to gender-affirming care can have life or death consequences for transgender people.^14^ Compared with their sample of campaigns for health care related to illness or injury, our results demonstrate that transgender people raising money for gender-affirming care are less successful at crowdfunding than the general population. Even with a lower average goal ($9,513 compared with $12,505), transgender recipients in our sample on average raised less than half than did the general population in Berliner and Kenworthy's sample ($1,296 compared with $3,033). Transgender recipients also raised a lower percentage of their goal than did the general population (24% compared with 41%).^28^

Results from our study indicate that social media has an impact on the total amount of money raised for medical care and the percentage of goal raised. It was not the number of *friends* a person has on Facebook that makes the difference for fundraising, but instead the number of *shares*. This result suggests that successful crowdfunding is related to having a large network of distant ties through which the fundraising page is shared, rather than fundraising success resulting from having a high number of close “friends” from which to solicit donations. Likewise, that the number of donors was significant in predicting the amount of money raised or percentage of one's goal suggests that having a large amount of small donations, rather than a small amount of large donations, predicts campaigns success. These findings are consistent with research on crowdfunding for entrepreneurial and cultural projects, such as developing new products or financing arts and film.^35,36^ In terms of crowdfunding medical care, that the number of times the campaign is shared on Facebook predicts raising more money and a larger percentage of one's goal has more serious consequences than not finishing a film or bringing a new gadget to market.

The modest successes in crowdfunding gender-affirming care are not evenly distributed. Although few campaigns were even moderately successful in reaching their goal, trans medical crowdfunding does not work the same for all recipients. First, older recipients were able to raise more funds than younger recipients. This may be a result of people older than their early 20s having greater social capital in terms of access to wealth in their networks. As a whole, the transgender population is disproportionately likely to be of low income, earn lower wages,^27^ and face discrimination in education and employment.^4^ Transgender youth in particular experience a number of barriers to socioeconomic mobility and may be financially dependent on adults who do not support their gender identity and/or medical gender affirmation.

Second, although on average trans women raised a similar amount of money to trans men ($1,312 and $1,298, respectively), they raised a lower average percentage of their goal (16% and 28%, respectively). When taking into account the Facebook shares and Facebook friends, trans men overall raised more money and a higher percentage than trans women. These findings may be reflective of the relative degree of social privilege afforded to some (passing, normatively masculine) transgender men through sexism^37^ and transmisogyny.^38^ However, when comparing trans men and trans women with an equal number of Facebook shares, trans women raised more money. That trans women with more Facebook shares raised more money than trans men who had the same number of shares is likely reflective of the fact that trans women are usually raising money for much more costly procedures,^26^ itself a manifestation of transmisogyny.

There are several limitations to our analysis. In particular, constructing a data set using material available on the web is itself limited to the information available on the page. We are missing data that could help explain campaign efficacy and the behavior of individuals contributing to a campaign. For example, the web site cannot tell us anything about the relationship between a recipient and those donating to their campaign that could indicate their location in the recipients' social network. We also cannot know what proportion of donations to a campaign comes from within the transgender community. Because our data set was cross-sectional, we were unable to track crowdfunding behavior and campaign success over time. Likewise, because several variables (e.g., gender identity, race, purpose of campaign) were created by assessing the campaign page, it is possible that we introduced error in our assignment of these variables. It was surprising that race was not significant in any of the models. This result could be due to the imperfect manner in which it was coded or because relatively few trans people of color prioritize web-based crowdfunding to meet their out-of-pocket costs. Finally, which transgender people utilize web-based crowdfunding is not random, and our results should not be interpreted as generalizable to other transgender people or to crowdfunding as a whole.

Future research should continue to track the scope and efficacy of medical crowdfunding for illness or injury as well as for gender-affirming care. Partnerships with crowdfunding web sites could allow for the construction of a longitudinal data set. In addition, further research should utilize interview or survey-based methods to analyze crowdfunding motivations, behaviors, and outcomes from the perspective of both recipients and contributors. Finally, given that the policy environment for insurance coverage for gender-affirming care is constantly changing, future research should consider the effects of these policies on crowdfunding practices; for instance, if crowdfunding shifts to funding co-pays and deductibles for gender-affirming care rather than the full costs of surgery. Research should continue to investigate the impacts of these policy changes on trans crowdfunding behaviors, access to health care, and health outcomes.

Medical and mental health providers serving transgender communities frequently encounter patients who turn to crowdfunding in the face of massive out-of-pocket expenses. Our research demonstrates that few of these patients will be successful in using crowdfunding to pay for their medical gender affirmation. With the continued erosion of the social safety net, particularly in the United States, the responsibility to pay for medically necessary care is being redistributed to vulnerable people's social networks. In essence, paying for health care becomes a popularity contest in which those with larger social networks come out on top. In our data, young transgender men in the United States are not only the most common recipients of medical crowdfunding but they are also more successful in harnessing the power of their social networks to pay for their care. Transgender medical crowdfunding in the United States is likely to expand in scope as legal protections for transgender health under the ACA are increasingly dismantled.^39^ Inequalities in medical crowdfunding for gender-affirming care may have life and death consequences for transgender individuals who are struggling to survive and thrive in the face of stigma, discrimination, and violence at multiple levels of society.

## Footnotes

^a^ It is possible that a higher percentage of people crowdfunding on the site understand their gender as something besides trans man or trans woman (e.g., genderqueer or nonbinary) but choose not to indicate it. In the U.S. Trans Survey, the largest nationally representative sample of trans and gender-variant people in the United States, 35% of respondents (*n*=27,715) selected “nonbinary” when given the choice to select between *woman*, *man*, *trans woman (MTF)*, *trans man (FTM)*, *nonbinary/genderqueer*, and *crossdresser.*

^b^ Nearly all fundraisers specifically stated their current gender identity and sex assigned at birth in their campaign narrative. Only seven fundraisers (1.7%) were unable to be coded for gender identity based on the information in the page and four (1%) were unable to be coded for sex assigned at birth. Whether or not the recipient was a person of color was more difficult to code. An imperfect solution was to code “yes, stated,” “yes, guessed,” “no, stated,” “no, guessed,” and “unclear/insufficient data” based on the text, photos, and name of recipient. These were then collapsed into a binary variable. Coding for race in this way is undoubtedly limiting and problematic but nevertheless important in order to identify potential racial inequalities in crowdfunding. It is possible that the proportion of people of color in the sample is over- or underestimated.

## Abbreviations Used

ACA: Affordable Care Act
FTM: female to male
MTF: male to female
